# Prussian Blue Nanoparticles Stabilize SOD1 from Ubiquitination‐Proteasome Degradation to Rescue Intervertebral Disc Degeneration

**DOI:** 10.1002/advs.202105466

**Published:** 2022-02-06

**Authors:** Tangjun Zhou, Xiao Yang, Zhiqian Chen, Yangzi Yang, Xin Wang, Xiankun Cao, Chen Chen, Chen Han, Haijun Tian, An Qin, Jingke Fu, Jie Zhao

**Affiliations:** ^1^ Shanghai Key Laboratory of Orthopedic Implants Department of Orthopedics Ninth People's Hospital Shanghai Jiaotong University School of Medicine 639 Zhizaoju Road Shanghai 200011 P. R. China

**Keywords:** discography contrast agent, intervertebral disc degeneration, mitochondrial structure, Prussian blue nanoparticles, reactive oxygen species, superoxide dismutase 1, ubiquitin‐proteasome degradation

## Abstract

Discography often destroys the hypoxic environment in the intervertebral disc and accelerates intervertebral disc degeneration (IVDD). Therefore, it often fails to meet the requirements for application in clinical practice. This technology mainly increases the reactive oxygen species (ROS) in the IVD. As so, it is particularly critical to develop strategies to avoid this degeneration mechanism. Prussian blue nanoparticles (PBNPs) are found to enhance development under magnetic resonance T1 and have antioxidant enzyme activity. The key results of the present study confirm that PBNPs alleviate intracellular oxidative stress and increase the intracellular activities of antioxidant enzymes, such as superoxide dismutase 1 (SOD1). PBNPs can rescue nucleus pulposus cell degeneration by increasing oxidoreductase system‐related mRNA and proteins, especially by stabilizing SOD1 from ubiquitination‐proteasome degradation, thus improving the mitochondrial structure to increase antioxidation ability, and finally rescuing ROS‐induced IVDD in a rat model. Therefore, it is considered that PBNPs can be a potential antioxidation‐protective discography contrast agent.

## Graphical abstract

Antioxidant enzyme‐like Prussian blue nanoparticles (PBNPs) can scavenge extracellular reactive oxygen species (ROS), inhibit the ubiquitin‐proteasome degradation of superoxide dismutase 1 in the cytoplasm to eliminate intracellular ROS, and

activate the transcription of antioxidant enzymes. Under these three key mechanisms, PBNPs can eventually activate nucleus pulposus cell viability, reconstruct the extracellular matrix of nucleus pulposus, rescue ROS‐mediated intervertebral disc degeneration.

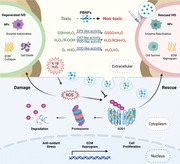

## 1 Introduction

The Global Burden of Diseases, Injuries, and Risk Factors Study (GBD) published in 2020 stated that low back pain (LBP) caused by intervertebral disc degeneration (IVDD) is one of the top ten causes of disability among 368 diseases.^[^ ^1^ ^]^ In China, the prevalence and years lived with disability (YLDs) rate for LBP slightly decreased from 1990 to 2016. However, the total number of individuals and number of YLDs increased. In fact, LBP is the second leading cause of YLD burden in China.^[^ ^2^ ^]^

Oxidative stress (OS) is an important factor in the molecular mechanism of IVDD. Nucleus pulposus cells (NPCs) in the intervertebral disc (IVD) are in an almost sealed hypoxic environment. However, after any minor damage to the intervertebral disc, the surrounding environment of NPCs becomes relatively hyperoxic, and reactive oxygen species (ROS), including superoxide (O_2_^•−^), hydroxyl (^•^OH), and peroxynitrite (ONOO^−^) increase, which results in the activation of inflammatory pathways, followed by cell apoptosis and inhibition of cell proliferation.^[^ ^3^ ^]^ Type II collagen (COL2) of the NPCs gradually decreases, while type I collagen (COL1) gradually increases, and aggrecan decomposition leads to decreased osmotic pressure in the IVD, followed by dehydration of the IVD matrix and increased outflow of matrix molecules.^[^ ^4^ ^]^ Subsequently, and along with the development of cavities and fissures of the nucleus pulposus and annulus fibrosus, there are a series of disc degeneration manifestations such as disc herniation, spinal canal stenosis, and nerve compression. Therefore, inhibition of OS, a key initial tracker, is an important strategy for preventing IVDD.

The clinical diagnosis of IVDD is based on magnetic resonance imaging (MRI), and for the accurate identification of discogenic LBP, multiple disc herniation, and nerve root symptoms, MRI of the IVD is often used clinically.^[^ ^5^ ^]^ However, this technology has a clear disadvantage: IVD puncture and the injection of the contrast agent will damage the nucleus pulposus and cause further degeneration. Contrast agent injection with puncture destroys the original intact structure of the annulus fibrosus, increases the pressure in the intervertebral disc,^[^ ^6^ ^]^ and causes the outflow of extracellular matrix and the influx of inflammatory factors; at the same time, the implantation of nonionic contrast agents (iopamidol and iohexol) further inhibits the proliferation of NPCs and annulus fibrosus cells and promotes apoptosis.^[^ ^7^ ^]^ Moreover, ionic contrast agents (e.g., ioxitalamate and indigocarmine) are toxic to cells,^[^ ^8^ ^]^ and this type of contrast agent can only be detected on radiography and computed tomography (CT), not on MRI. Therefore, images are often less detailed than enhanced MRI. However, the clinical application of discography is indispensable. Therefore, the development of a less toxic contrast agent used in MRI that can protect NPCs and the extracellular matrix has become crucial for both basic research and clinical applications.

Several studies have shown that Prussian blue nanoparticles (PBNPs) have a variety of similar peroxidase (POD), catalase (CAT), and superoxide dismutase (SOD) enzymes that can remove O_2_^•−^ and hydrogen peroxide (H_2_O_2_) ROS in neutral environments, such as the cytoplasm, and ^•^OH in acidic environments, such as the lysosome.^[^ ^9^ ^]^ Furthermore, PBNPs have been shown to eliminate H_2_O_2_, ^•^OH, ONOO^−^, and O_2_^•−^ thereby inhibiting the inflammatory pathways MAPK/ERK and NF‐*κβ* and reduce nerve cell apoptosis in a rat model of ischemic stroke.^[^ ^10^ ^]^ Moreover, PBNPs cleared ROS in myeloid leukemia cells (K562), downregulated NFE2, upregulated GATA1, and differentiated cells into terminal erythrocytes.^[^ ^11^ ^]^ PBNPs also cleared ROS in the intestine and decreased intestinal lesions in a dextran sulfate sodium (DSS)‐induced enteritis mouse model.^[^ ^12^ ^]^ More interestingly, PBNPs enhanced the signal of MRI T1 images.^[^ ^13^ ^]^ Considering ROS clearing as a route to rescue IVD degeneration, PBNPs have the potential to become antioxidant agents in discography. However, evidence of a direct molecular mechanism for the regulation of the redox environment inside and outside the NPCs is still lacking. Whether PBNPs can affect key anti‐degenerative factors or pathways in NPCs, and the mechanism by which these effects are produced is still unclear. In the present study, we provide new and more direct evidence based on protein interactions.

## 2 Results

### 2.1 Characterization of PBNPs

**Figure 1:**
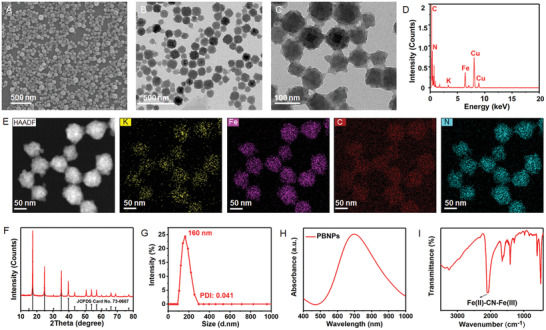
Characterization of as‐prepared PBNPs. A) SEM image of PBNPs showed the formation of uniform PBNPs with good monodispersity. B,C) TEM images of cube PBNPs with the average size of ≈80 nm. D) EDS of PBNPs confirmed PBNPs contained potassium (K), iron (Fe), carbon (C), and nitrogen (N) elements. E) Element mappings of K, Fe, C, and N elements in PBNPs. F) XRD pattern of the as‐prepared PBNPs and standard JCPDS Card No. 73‐0687. G) The average hydrodynamic particle diameter of PBNPs indicating good dispersion of these particles in aqueous media. H) UV–vis–NIR absorbance curve of PBNPs displayed a wide absorbance peak around 700 nm. I) FT‐IR spectrum of PBNPs showed an obvious characteristic peak at 2094 cm^−1^.

In this work, polyvinylpyrrolidone (PVP)‐modified PBNPs were prepared using a simple hydrothermal procedure according to the literature.^[^ ^14^ ^]^ Scanning electron microscopy (SEM) images showed the formation of uniform PBNPs with good monodispersity (**Figure** **1**A). Transmission electron microscopy (TEM) images indicated that the morphology of the prepared PBNPs was spherical, and the average size of the PBNPs was ≈80 nm (Figure 1B,C). The energy‐dispersive X‐ray spectrum (EDS) confirmed the presence of potassium (K), iron (Fe), carbon (C), and nitrogen (N) with no impurities (Figure 1D). Element mapping further demonstrated that K, Fe, C, and N elements were distributed in the framework of PBNPs with high uniformity (Figure 1E). In addition, the crystal structures of the prepared PBNPs were characterized using X‐ray powder diffraction (XRD). As shown in Figure 1F, the diffraction peaks at the 2*θ* values of 17.5°, 24.8°, 35.4°, and 39.8° were assigned to the (200), (220), (400), and (420) crystal planes of Fe cyanide (JCPDS card No. 73‐0687). The average hydrodynamic particle diameter of PBNPs was determined to be 160 nm with a narrow size distribution (polydispersity index (PDI): 0.041), indicating good dispersion of these particles in aqueous media (Figure 1G). The UV–vis–near infrared (NIR) absorbance spectrum of PBNPs displayed a wide absorbance peak at ≈700 nm due to the intermetallic charge transfer from Fe(II) to Fe(III) (Figure 1H).^[^ ^15^ ^]^ Fourier transform infrared (FT‐IR) spectroscopy showed an obvious characteristic peak at 2094 cm^−1^ (Figure 1I), which could be attributed to the cyano group (–CN–) stretching in the Fe(II)–CN–Fe(III) bond of PBNPs.^[^ ^16^ ^]^ In addition, the characteristic peak at 1655 cm^−1^ was assigned to the carbonyl group (C═O) of PVP molecules, indicating the presence of PVP in the PBNPs.

### 2.2 Multiple Enzyme‐Like Activities of PBNPs

**Figure 2:**
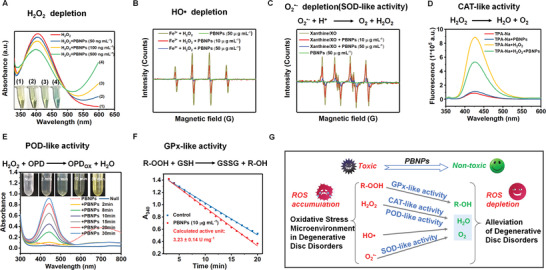
Multiple enzyme‐like activities of PBNPs. A) H_2_O_2_ depletion of PBNPs determined by titanium sulfate method. B) CAT‐like activity and H_2_O_2_ depletion of PBNPs determined by disodium terephthalate (TPA‐Na). C) •OH depletion capacity of PBNPs determined by EPR. D) SOD‐like activity and O_2_^•−^ depletion of PBNPs determined by EPR. E) POD‐like activity of PBNPs using OPD as the chromogenic substrate. F) GPX‐like activity of PBNPs determined by Glutathione Peroxidase Assay Kit with NADPH. G) Illustration of the multiple enzyme‐like activities of PBNPs, which are expected to scavenge the ROS in cells and alleviate the harsh oxidative stress microenvironment in degenerative disc disorders. The experiments above were repeated three times.

ROS have been identified as essential mediators during the occurrence and progression of IVDD.^[^ ^17^ ^]^ PBNPs may protect cells from ROS‐induced oxidative stress by scavenging a variety of these radicals, including H_2_O_2_, •OH, and O_2_^•−^. The H_2_O_2_ scavenging capacity of PBNPs was initially explored using titanium sulfate, which could react with H_2_O_2_ to generate a spectrophotometrically yellow peroxide–titanium complex with a characteristic peak at 415 nm. As shown in **Figure** **2**A, the absorbance at 415 nm decreased with the addition of PBNPs. In addition, a further decrease in the absorbance at 415 nm was observed when the concentration of PBNPs was increased, demonstrating the PBNP concentration‐dependent depletion of H_2_O_2_ (Figure S1A, Supporting Information).

CAT or CAT‐mimics catalyze the decomposition of H_2_O_2_ to generate O_2_ and H_2_O, protecting living organisms from H_2_O_2_ induced oxidative damage. To further explore the CAT‐like activity of PBNPs, H_2_O_2_ decomposition was evaluated using disodium terephthalate (TPA‐Na).^[^ ^18^ ^]^ The nonfluorescent TPA‐Na could convert H_2_O_2_ into a fluorescent indicator with a characteristic peak at 425 nm. As shown in Figure 2B, the fluorescence intensity at 425 nm was significantly decreased in the presence of PBNPs, demonstrating their effective CAT‐like activity. The •OH depletion capacity of PBNPs was then evaluated by electron paramagnetic resonance (EPR) using 5,5‐dimethyl‐1‐pyrroline N‐oxide (DMPO) as the spin probe. As demonstrated in Figure 2C, •OH was initially produced by the Fe^2+^/H_2_O_2_ reaction system, showing distinct and characteristic •OH signals in the EPR. The addition of PBNPs resulted in a steep and concentration‐dependent decline in the •OH signal intensity, suggesting a significant depletion of •OH after low‐dose PBNPs treatment.

SOD is a classical antioxidant enzyme that converts O_2_^•−^ into H_2_O_2_ and O_2_. To investigate the SOD‐like activity of PBNPs, an O_2_^•−^‐generating xanthine/xanthine oxidase (XO) system was used, and the O_2_^•−^ scavenging ability of PBNPs was evaluated by EPR using DMPO as the spin probe. A strong and characteristic signal peak of DMPO/O_2_^•−^ appeared in the xanthine/XO system (Figure 2D). The signal intensity displayed a distinct decline upon the addition of PBNPs (10 µg mL^−1^). When the PBNPs concentration was further increased to 50 µg mL^−1^, a steeper decline in the signal intensity was observed, indicating the excellent SOD‐like activity of PBNPs for the scavenging of O_2_^•−^.

POD is another antioxidant enzyme that can detoxify H_2_O_2_ into H_2_O.^[^ ^19^ ^]^ Therefore, the POD‐like activity of PBNPs was assessed using *o*‐phenylenediamine (OPD) as the chromogenic substrate. Upon the addition of PBNPs, time‐dependent colorization of OPD was observed (Figure 2E). The colorization was further confirmed by its maximum characteristic absorbance at 442 nm, which increased as the reaction proceeded. Glutathione peroxidase (GPX) catalyzes the reduction of peroxides in the presence of cellular glutathione (GSH).^[^ ^20^ ^]^ The GPX‐like activity of PBNPs, determined using a GPX assay kit with NADPH, was ≈3.23 U mg^−1^ (Figure 2F). The multiple enzyme‐like activity and the excellent ROS scavenging capacity of PBNPs evidenced above are thus expected to alleviate the harsh OS microenvironment in degenerative disc disorders and act as a promising antioxidant for IVDD treatment (Figure 2G).

### 2.3 PBNPs Alleviate Intracellular OS to Rescue the Degeneration of NPCs

**Figure 3:**
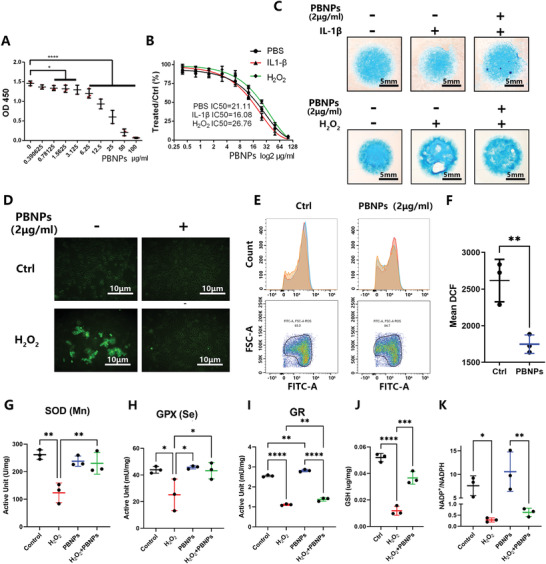
Cytological phenomenon after treatment of NPCs with PBNPs. A) Cell viability of NP cells tested by CCK8 after gradient concentrations of PBNPs (0.39–100 µg mL^−1^) treatment for 24 h. The *Y*‐axis is the OD 450 value. B) After IL1‐*β* (10 ng mL^−1^), H_2_O_2_ (0.6 × 10^−3^%) stimulation, PBNPs (0.39–100 µg mL^−1^) was treated to NP cells for 24 hours. The *Y*‐axis is the ratio of the OD 450 value of PBNPs treatment groups to the control group. IC50 referred to the concentration of PBNPs. C) NP cells in high density culture was treated with IL‐1*β* (10 ng mL^−1^) or H_2_O_2_ (0.6 × 10^−3^%) and PBNPs (2 µg mL^−1^), strained Alcian blue in day 5. D) After H_2_O_2_ (0.6 × 10^−3^%) stimulation and PBNPs (2 µg mL^−1^) treatment, oxygen species (RS) in NP cells was detected via DCFH/DCF ROS detecting system. E) ROS in NP cells was detected by flow cytometry combined with DCFH/DCF ROS detecting system. F) Statistical chart of mean DCF according to flow cytometry. The significance of difference was tested with *t*‐test. G–K) Statistical chart shows active unit (U mg^−1^) of SOD(Mn), active unit (mU mg^−1^) of GPX(Se), active unit (mU mg^−1^) of GR, concentration of GSH (µg mg^−1^), ratio of NADP+ to NADPH after H_2_O_2_ (0.6 × 10^−3^%) stimulation and PBNPs (2 µg mL^−1^) treatment. The data were presented using mean ± S.D. * indicates *p* < 0.05, ** indicates *p* < 0.01, *** indicates *p* < 0.001. **** indicates *p* < 0.0001. The experiments above were repeated three times.

The cell viability of NPCs was tested using the cell counting kit 8 (CCK8) assay under gradient concentrations of PBNPs. The safe concentration of PBNPs was below 3.125 µg mL^−1^ (**Figure** **3**A). Interleukin (IL)1‐*β* and H_2_O_2_ were confirmed to induce inflammation and OS in NPCs, and gradient concentrations of PBNPs were then used to treat NPCs. In the OS environment, the half‐maximal inhibitory concentration (IC50) of PBNPs was significantly higher than that of the control group. However, in the inflammatory environment, the IC50 of PBNPs did not increase significantly (Figure 3A). Therefore, the safe concentration of PBNPs to NPCs was detected, and most cell experiments conducted in vitro thereafter used a concentration of 2 µg mL^−1^.

PBNPs were added to the high‐density culture of NPCs to detect their effect on the degeneration phenotype. IL1‐*β* groups induced an inflammatory response and H_2_O_2_ groups induced OS, resulting in a decrease in Alcian blue staining, which indicated a decrease in extracellular matrix (ECM) secretion from the NPCs. PBNPs at a concentration of 2 µg mL^−1^ rescued ECM secretion (Figure 3C). Therefore, PBNPs effectively rescued the ECM secretion induced by H_2_O_2_, indicating that PBNPs can reduce the degeneration phenotype of NPCs under OS.

Furthermore, the dichlorodihydrofluorescein‐diacetate (DCF‐DA) method was used to detect ROS in NPCs. The results showed that H_2_O_2_ induced an increase in the DCF of NPCs, indicating an increase in ROS and thus the success of the cell model. The use of 2 µg mL^−1^ PBNPs reduced the increase in ROS and rescued the cell model (Figure 3D). Furthermore, we used flow cytometry to detect ROS labeled by DCF in NPCs and found that the peak value of DCF green fluorescence (488 nm) shifted to the left after treatment with 2 µg mL^−1^ PBNPs (Figure 3E), indicating that ROS in NPCs were cleared. The mean DCF of NPCs was significantly lower than that of the control group (Figure 3F).

### 2.4 PBNPs Increase Intracellular GSH and the Activities of SOD, GPX, and Glutathione Reductase (GR)

As PBNPs were shown to increase extracellular SOD activity, this was detected. SOD with manganese (Mn) core (SOD(Mn)) decreased significantly under the stimulation of H_2_O_2_, and the subsequent addition of PBNPs significantly improved the activity of SOD(Mn) (Figure 3G). The activities of GPX (total and selenium core) and GR were also detected. The activity of total GPX in NPCs decreased under H_2_O_2_ stimulation, and the subsequent addition of PBNPs significantly increased the activity of total GPX (Figure S1B, Supporting Information) and GPX with selenium core (Figure 3H). Furthermore, the activity of GR in NPCs decreased with the addition of H_2_O_2_, and PBNPs significantly improved the activity of GR (Figure 3I).

Changes in the key compound GSH were detected. The test results showed that GSH decreased after H_2_O_2_ stimulation and that PBNPs increased GSH levels (Figure 3J). NADP^+^/NADPH was also detected as a side reaction and product. NADP^+^/NADPH decreased significantly under H_2_O_2_ stimulation, and there was an upward trend with the addition of PBNPs (Figure 3K).

### 2.5 PBNPs Rescue NPCs Degeneration Through Oxidoreductase System mRNAs and Proteins

**Figure 4:**
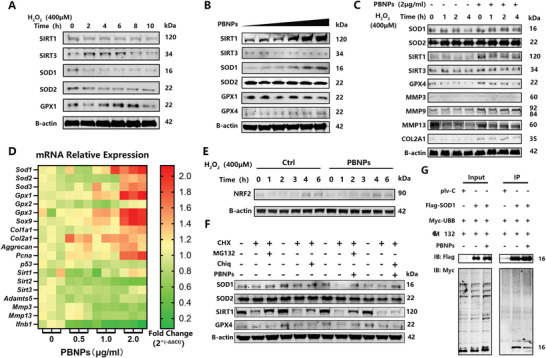
Intracellular molecular mechanism of PBNPs treated NPCs. A) Expression of SIRT1, SIRT3, SOD1, SOD2, GPX1, B‐actin protein from increasing time of H_2_O_2_ (0.6 × 10^−3^%) treated NP cells detected by WB. B) Expression of SIRT1, SIRT3, SOD1, SOD2, GPX1, GPX4, B‐actin protein from gradient concentrations of PBNPs (0.125–2 µg mL^−1^) treated NP cells detected by WB. C) Expression of SOD1, SOD2, SIRT1, SIRT3, GPX4, MMP3, MMP9, MMP13, COL2AL, B‐actin protein from increasing time of H_2_O_2_ (0.6×10^−3^%) and PBNPs (2 µg mL^−1^) treated NP cells detected by WB. D) Expression of *Sod1*, *Sod2*, *Sod3*, *Gpx1*, *Gpx2*, *Gpx3*, *Sox9*, *Col1a1*, *Col2a1*, *Aggrecan*, *Pcna*, *p53*, *Sirt1*, *Sirt2*, *Sirt3*, *Adamts5*, *Mmp3*, *Mmp13*, *Ifnb1* mRNA from gradient concentrations of PBNPs (0.5, 1, 2 µg mL^−1^) treated NP cells detected by PCR. Date showed as Fold Change (2^‐ΔΔCT^). E) Expression of NRF2 protein from increasing time of H_2_O_2_ (0.6 × 10^−3^%) and PBNPs (2 µg mL^−1^) treated NP cells detected by WB. F) Expression of SOD1, SOD2, SIRT1, GPX4, B‐actin protein from PBNPs (2 µg mL^−1^), CHX(50 × 10^−9^ m), MG132(10 × 10^−6^ m ), Chiq (25 × 10^−9^ m) treated NP cells detected by WB. G) Co‐IP assay of Flag‐SOD1, Myc‐UBB overexpressed 293T treated with MG132 and PBNPs. IB with Flag and Myc. The first line of WB records to control groups of 293T overexpressed plv‐C and Myc‐UBB treated with MG132. The experiments above were repeated three times.

Subsequently, we focused on the changes in protein expression in the oxidoreductase system. NAD‐dependent protein deacetylase sirtuin 1 (SIRT1), SOD1, and SOD2 decreased with increasing time of H_2_O_2_ stimulation, while SIRT3 and GPX1 increased and then decreased (**Figure** **4**A). *tert*‐butyl hydroperoxide (TBHP) was used to induce OS in a parallel test (Figure S1C, Supporting Information). With increasing concentrations of TBHP (0–50 × 10^−6^ m), the expression of SOD2 and GPX4 decreased, while that of SIRT3 increased slightly, and that of GPX1 and GPX3 showed an upward trend. Finally, we selected H_2_O_2_ as a stimulator because most of the results followed the changes in the OS cell model.^[^ ^21^ ^]^ We found that with increasing concentrations of PBNPs (0–2 µg mL^−1^) in NPCs, SIRT1, SOD1, and GPX4 also increased, while SIRT3 and GPX1 decreased slightly (Figure 4B). Furthermore, after H_2_O_2_ treatment with a time gradient of 0 to 4 h, the expressions of SOD1, SIRT1, SIRT3, and GPX4 decreased after the addition of H_2_O_2_; GPX4 expressions increased after the addition of PBNPs, while SOD2 expression showed no change (Figure 4C). We also detected the catabolism and anabolism markers of NPCs, matrix metalloproteinase family (MMP3/9/13), and COL2A1. After the addition of H_2_O_2_, the levels of MMP3/9/13 and COL2A1 decreased. After PBNPs addition, protein expression significantly increased (Figure 4C).

We found that PBNPs have a regulatory effect on oxidoreductase system proteins. However, it seems that this regulatory effect has different responses under different OSs. At the transcriptional level, we detected the mRNA expression of these proteins (Figure 4D; Figure S2, Supporting Information). We found that PBNPs could increase the expression of intracellular oxidoreductase mRNA, including *Sod1/2/3* and *Gpx1/3*, and decreased the expression of *Sirt1/2/3*. In the ECM anabolism of NPCs, *Col1a1*, *Col2a1*, and *Aggrecan* increased, while in ECM catabolism, *MMP*s and *Adamts5* decreased. Regarding cell proliferation markers, *PCNA* was upregulated, and *p53* was downregulated. The expression of the inflammatory factor interferon beta 1 (*Ifnb1*) was downregulated. Therefore, PBNPs can promote the anabolism of NPCs, inhibit catabolism, promote cell proliferation, and reduce the inflammatory response. More importantly, the changes in the expression of oxidoreductase system proteins can be partially explained by the alleviation of cellular transcription levels by PBNPs. However, the intermediate pathway of this regulation needs to be further explored, as changes in *SOD* mRNA levels remain unclear.

RNA sequencing of NPCs treated with PBNPs was performed to explore the pathway regulating mRNA transcription of *SOD*. Classical pathways, such as Ras, p53, PI3K, Akt, and AMPK, have been shown to play important roles, and it was verified that PBNPs can enhance the expression of ECM genes, such as *Col2a1* and *Itga7*. Genes such as *Ntrk2* and *Cxcr4*, which promote cell proliferation and anti‐apoptosis, are also involved. Furthermore, the expression of ubiquitinated genes, such as those in the ubc13‐uev1a pathway, takes part in the reaction to PBNP treatment (Figure S3, Supporting Information). However, we found no direct evidence that changes in these pathways were related to an increase in SOD1 mRNA expression, protein content, and activity.

### 2.6 PBNPs Inhibit SOD1 Degradation through the Ubiquitin‐Proteasome Pathway

In the trials described in the previous sections, PBNPs were shown to increase SOD1 at both the mRNA and protein levels, but the molecular mechanism remains unclear. More importantly, a negative result showed that nuclear factor‐erythroid 2‐related factor 2 (NRF2) increased under H_2_O_2_ stimulation with increasing time, and PBNPs did not change this upward trend (Figure 4E). NRF2 is an important binding factor of antioxidant responsive element (ARE2),^[^ ^22^ ^]^ which mediates the transcription of antioxidant stress proteins, such as that in the SOD family.^[^ ^23^ ^]^ Therefore, we further explored whether PBNPs have a direct mechanism to regulate SOD1.

Protein degradation was first considered. We used cycloheximide (CHX) to inhibit protein synthesis, MG132 (Z‐LLL‐CHO) to inhibit proteasome, chloroquine (Chiq) to inhibit lysosomes, and added PBNPs. We found that SOD1 was mainly degraded by proteasomes. After addition of CHX, Chiq, and PBNPs, the content of SOD1 protein was significantly higher than that obtained without the addition of PBNPs. Moreover, there was no significant change in SOD1 protein content after the addition of CHX, MG132, and PBNPs, indicating that PBNPs can inhibit the proteasomal degradation of SOD1. For SOD2, the abundance was so high that there was no significant protein degradation after the addition of CHX. For GPX4, no degradation was detected through the proteasome or lysosomal pathways. In addition, we found that SIRT1 could be degraded through the proteasome and lysosomal pathways, mainly by the first. After the addition of CHX, Chiq, and PBNPs, the protein was still degraded, indicating that PBNPs could mediate the proteasomal degradation of SIRT1 (Figure 4F).

Furthermore, we used co‐immunoprecipitation (Co‐IP) to verify if PBNPs could inhibit SOD1 degradation through the proteasome. We found that SOD1 bound to polyubiquitin‐b (UBB) but this binding was decreased when PBNPs were added, revealing that PBNPs can inhibit the ubiquitination modification of SOD1. In conclusion, these trials revealed that PBNPs can inhibit the ubiquitination modification of SOD1 to inhibit the degradation of this enzyme through the proteasome pathway (Figure 4G).

### 2.7 PBNPs Improve Mitochondrial Structure under OS by Interacting with SOD1 in NPCs

**Figure 5:**
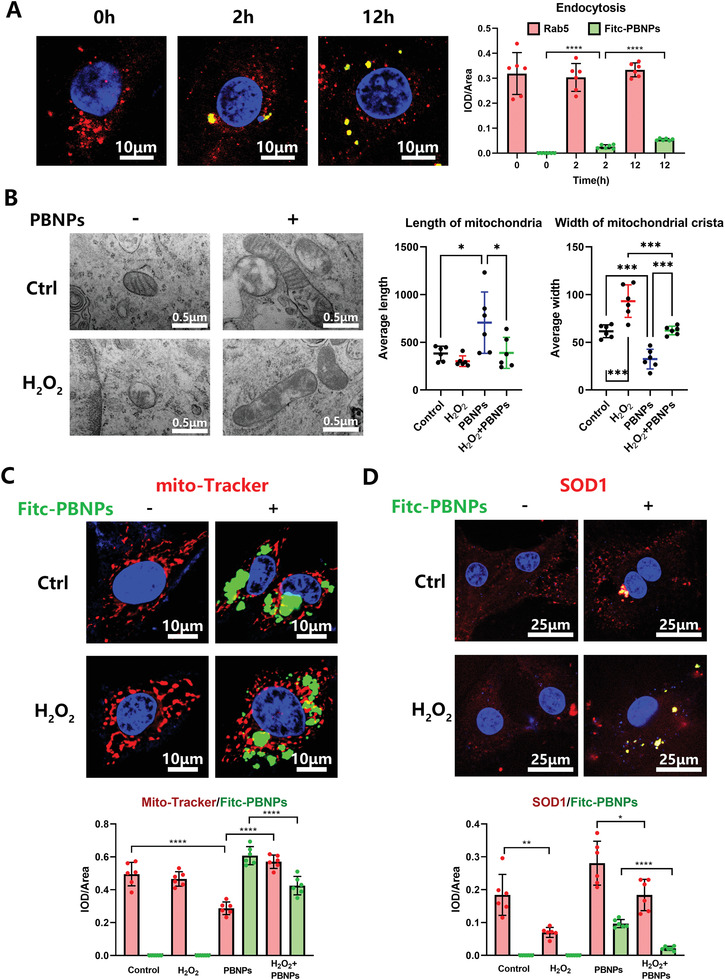
Localization of PBNPs in NPCs. A) Fitc‐PBNPs was treated to NP cells with increasing time and colocalization of Rad5 with Fitc‐PBNPs was detected by Confocal. Statistical chart shows IOD/area of red and green fluorescence. B) TEM images of intracellular mitochondria in NP cells, after H_2_O_2_ (0.6 × 10^−3^%) stimulation and PBNPs (2 µg mL^−1^) treatment. Statistical chart shows length of mitochondria and width of mitochondria crista. C) NP cells, after H_2_O_2_ (0.6 × 10^−3^%) stimulation and Fitc‐PBNPs (2 µg mL^−1^) treatment was strained with mito‐Tracker. Statistical chart shows IOD/area of red and green fluorescence. D) After H_2_O_2_ (0.6 × 10^−3^%) stimulation and Fitc‐PBNPs (2 µg mL^−1^) treatment, colocalization of SOD1 with Fitc‐PBNPs was detected by Confocal. Statistical chart shows IOD/area of red and green fluorescence. The data were presented using mean ± S.D. * indicates *p* < 0.05, ** indicates *p* < 0.01, *** indicates *p* < 0.001, **** indicates *p* < 0.0001. The experiments above were repeated six times.

The PBNPs were labeled with fluorescein isothiocyanate (FITC). After only 2 h of treatment, PBNPs colocalized with Rab5, which is an early endocytosis marker,^[^ ^24^ ^]^ indicating that PBNPs can be endocytosed by NPCs (**Figure** **5**A), and endocytosis peaked at 12–24 h (Figure S4A, Supporting Information). The excretion of PBNPs was observed at 24 h, and PBNPs partially excreted cells at 48 h (Figure S4B, Supporting Information). The above results showed that PBNPs can shuttle inside the cell by endocytosis and that PBNPs only exist in the cytoplasm and around the mitochondria.

TEM images showed that mitochondria in the H_2_O_2_ group had more vacuoles, less crista, and smaller size. Moreover, the length of mitochondria had a decreasing tendency and the width of the mitochondrial crista was significantly less than that observed in the control group. Further, the length of mitochondria increased and the width of mitochondrial crista decreased after PBNPs treatment with or without H_2_O_2_ treatment (Figure 5B). Therefore, PBNPs can increase the size of mitochondria and the number of mitochondrial crista to rescue unhealthy mitochondria under OS.

Intracellular mitochondria were labeled with a MitoTracker probe with red fluorescent CMXRos. PBNPs were located around the mitochondria, but there was no fusion with them. The intensity of CMXRos in the H_2_O_2_ group did not differ from that in the control group. Under PBNPs treatment, the intensity of CMXRos was lower, indicating fewer mitochondria (Figure 5C). SOD1 colocalized with PBNPs and could be increased by this treatment with or without H_2_O_2_ treatment (Figure 5D). These results showed that PBNPs can effectively restore the functional structure of mitochondria by stabilizing SOD1 to induce antioxidant stress. Similar to SOD1, the expression of SIRT1 and GPX4 under OS was downregulated to varying degrees in NPCs, and PBNPs recovered it as shown by immunofluorescence (IHF) (Figure S4C, Supporting Information).

### 2.8 PBNPs Rescue Rat IVD through Stabilizing SOD1 from Ubiquitination Degradation

**Figure 6:**
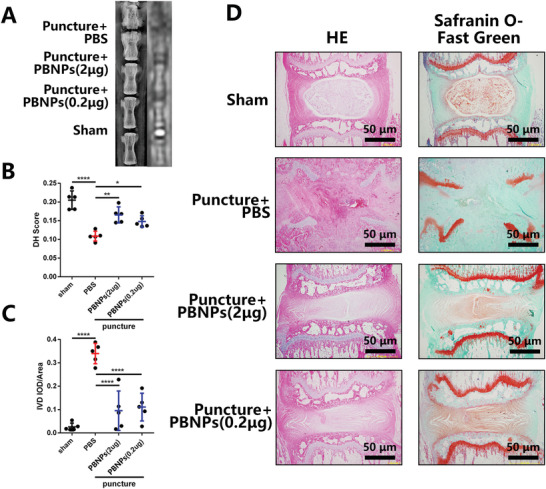
PBNPs rescue IVDD in rats. A) X‐ray and MRI of rat tail including puncture group, puncture with PBNPs (2 µg) injection group, puncture with PBNPs (0.2 µg) injection group and sham group. Rats were sacrificed 4 weeks after injection. B,C) Statistical chart of DH and ratio of IOD of IVD to IVD area. D) HE and Safranin O‐Fast Green staining. The data were presented using mean ± S.D. * indicates *p* < 0.05, ** indicates *p* < 0.01, **** indicates *p* < 0.0001. The experiments above were repeated five times.

A rat caudal puncture IVD model was used for the in vivo study. PBNPs were administered immediately after injection to simulate the circumstances of discography in the clinic. After local injection of PBNPs into the IVD, no lesions were found in the heart, liver, spleen, lung, kidney, or other important organs of rats (Figure S5, Supporting Information). After proving the safety concentration for major organs, we focused on IVDD (**Figure** **6**; Figure S6A,B, Supporting Information). IVDD in the punctured group was the heaviest, and it was significantly relieved after PBNPs administration; no significant differences were found among the PBNP treatments at different concentrations (Figure 6). As detected by X‐ray, the intervertebral height score (DH score) of the puncture+PBS group was significantly lower than that of the sham group, the osteophyte of the puncture+PBS group was significantly increased (Figure 6A,B), and the Integrated Optical Density/area (IOD/area) of intervertebral space in the puncture+PBS group increased significantly (Figure 6A,C). In contrast, the DH score of the puncture+PBNPs group increased significantly, and X‐ray showed that osteophytes in the intervertebral space decreased significantly (Figure 6A,B). The IOD/area of the intervertebral space decreased significantly (Figure 6A,C). From the perspective of imaging detection, PBNPs could reduce the puncture‐induced degeneration of the caudal IVD in rats.

Furthermore, histological examination of the rat IVD showed that in the puncture+PBS group, the intervertebral space collapsed, and there was severe fibrosis in the intervertebral space and in the upper and lower cartilage endplates; the growth plate was also destroyed. In the puncture+ PBNPs group, the annulus fibrosus remained at the periphery of the intervertebral space, the boundary between the annulus fibrosus and the nucleus pulposus was not clear, and the center of the intervertebral space remained the nucleus pulposus under red safranin staining (Figure 6D). Nevertheless, the tissue structure of the nucleus pulposus differed from that of the sham operation group, showing slight degeneration. For rats sacrificed 2 weeks after PBNPs injection, the same tendency was observed (Figure S7, Supporting Information).

**Figure 7:**
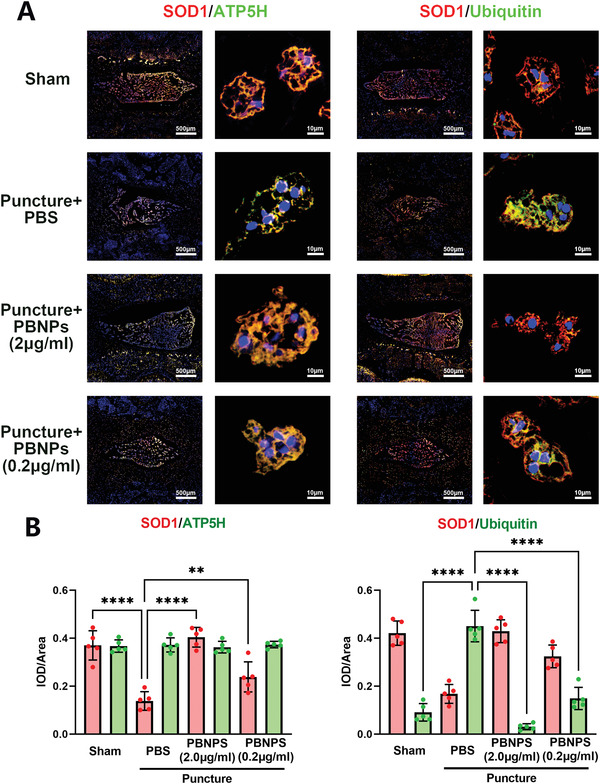
Molecular mechanism of PBNPs rescued IVDD in rats. A) Colocalization of SOD1 with ATP5H and Ubiquitin in sham group, puncture group, puncture with PBNPs (2 µg) injection group, puncture with PBNPs (0.2 µg) injection group via IHF. B) Statistical chart shows IOD/area of red and green fluorescence. The data were presented using mean ± S.D. ** indicates *p* < 0.01, **** indicates *p* < 0.0001. The experiments above were repeated five times.

Subsequently, we labeled mitochondria in NPCs using ATP5H, targeted to Complex V, which is an important marker of oxidative phosphorylation,^[^ ^25^ ^]^ and found that SOD1 colocalized with mitochondria. At the same time, it was found that SOD1 in the puncture group was downregulated, while SOD1 was upregulated after the addition of PBNPs in a concentration‐dependent manner. SOD1 and ubiquitin were partially colocalized. In the puncture group, SOD1 was downregulated, and ubiquitin was upregulated. PBNPs reduced the expression of SOD1 and downregulated ubiquitin in a concentration‐dependent manner (**Figure** **7**). Aggrecan and COL2A1 were rescued by PBNPs (Figure S8A, Supporting Information), which could also rescue the low expression of GPX1 and SIRT1 caused by puncture (Figure S8B, Supporting Information). Taken together, PBNPs can stabilize SOD1 by inhibiting the ubiquitination of SOD1 in or around mitochondria in NPCs, thereby enhancing the antioxidant stress ability of NPCs, and finally increasing the extracellular matrix to inhibit IVDD.

Interestingly, MRI verified that PBNPs can display high signal on T1 and low signal on T2, which was related to its concentration gradient (Figure S6C, Supporting Information). The T1 relaxation time and T1 relaxation rate tested by T1 mapping image were showed in statistical chart (Figure S9, Supporting Information). The T1 relaxation rate of PBNPs (100 µg mL^−1^, 0.505 ± 0.025 s^–1^), which was significantly lower than Gd‐DTPA (100 µg mL^−1^, 0.826 ± 0.038 s^–1^). Further, T1 relaxation rate of PBNPs increased with concentration dependence. This indicated that PBNPs can enhance MRI T1 image which is lower than Gd‐DTPA. However, the in vivo imaging application needs to be further studied.

## 3 Discussion

The extracellular effect of PBNPs has been partially clarified, as it mainly acts on the elimination of ROS. The key mechanism of this effect is that they are similar for POD, CAT, and SOD, which will form nontoxic and harmless H_2_O, OH, and O_2_. Many in vivo and in vitro studies have shown that PBNPs can protect cells through anti‐inflammatory pathways.^[^ ^26^ ^]^

Similar to previous studies, our study also verified the similar role of PBNPs in SOD, CAT, and POD activities;^[^ ^9^ ^]^ however, results for GPX activity in vitro were new. We found that PBNPs can scavenge R‐OOH and produce glutathione disulfide (GSSG) and R‐OH by catalytically consuming GSH.

The nucleus pulposus environment is hypoxic. NPCs in anaerobic metabolism break due to rupture of the fibrous ring. When oxygen enters the nucleus pulposus, it begins aerobic metabolism, which produces ROS and further destroys NPCs.^[^ ^27^ ^]^ Therefore, we thought of using PBNPs for clearing ROS, as reported for decreasing hyperthermia‐induced side effects in tumor photothermal therapy,^[^ ^26^ ^]^ and looked for possible degeneration and rescue. First, we defined the IC50 of PBNPs, determined its nontoxic concentration, and verified that PBNPs can reduce the viability of NPCs under H_2_O_2_‐induced OS. As vitality can be rescued, it is not surprising that the high‐density culture experiment of NPCs could also rescue the differentiation ability of NPCs under OS induced by H_2_O_2_. This phenomenon can possibly be explained by the results obtained from labeling ROS with DCFH‐DA, as PBNPs effectively removed ROS from NPCs. In cells, the conversion of reduced GSH and oxidized GSSG depends on the activities of GPX and GR. GSH and H_2_O_2_ react with GSSG and H_2_O through GPX to remove H_2_O_2_ and generate GSSG.^[^ ^28^ ^]^ GR can use NADPH to regenerate oxidized GSSG into GSH, with NADP^+^ as the by‐product.^[^ ^29^ ^]^ Not surprisingly, we detected that GPX, GR, GSH, and NADP^+^/NADPH in PBNP‐treated NPCs increased to varying degrees. The above changes in enzyme activities and redox products maintain NPCs in a reducing environment and contribute to their successful resistance to ROS. More importantly, we found that PBNPs can improve the activity of intracellular SOD and that SOD1 plays a key role in the anti‐ROS mechanisms of NPCs. This discovery prompted us to conduct a trial that allowed obtaining the results described below, which are among the most important arguments of the present study.

PBNPs scavenge ROS outside the cell. How do they scavenge ROS within cells? Is this ability crosslinked with extracellular scavenging? To answer these questions, we first investigated whether and how PBNPs enter the cells and identified endocytosis as one possible mechanism. We found that PBNPs could interact with Rab5, the key marker in the early phase of endocytosis^[^ ^24^ ^]^ and a mediator of the endocytosis of PBNPs. Endocytosed PBNPs can diffuse around the mitochondria without directly entering them, as observed in the TEM and MitoTracker tests. Therefore, we pretreated NPCs with PBNPs for 24 h to verify that the NPCs engulfed the PBNPs. Then, OS inducers, including H_2_O_2_ and TBHP, were added to detect the main possible downstream molecular pathways to exclude the direct extracellular scavenging effect of PBNPs on H_2_O_2_ and TBHP. Interestingly, the addition of PBNPs alone increased the protein content of SOD1 and SIRT1 in cells, indicating the direct effect of PBNPs on intracellular protein expression. Then, after adding the OS inducer, various antioxidant enzymes in cells were consumed, and the protein content of SOD1/2, SIRT1/3, and GPX3/4 in the group pretreated with PBNPs was increased, suggesting that the intracellular reducing environment caused by PBNPs can effectively confer resistance to OS. However, a similar role of PBNPs in SOD, CAT, POD, and GPX has been previously reported in vitro.^[^ ^9^ ^]^ Our study found that PBNPs can directly enhance the activity and content of antioxidant enzymes in cells, especially SOD1, which has not been detected or explained in previous studies. Hence, we studied this further.

Regarding the upstream molecular mechanism of directly increasing protein content, we first needed to consider significant changes at the mRNA level. Under a gradient concentration of PBNPs, changes occurred in the mRNA expression of many antioxidant enzymes, particularly the upregulation of the SOD family and GPX1/3 and the downregulation of SIRT2. This can partly explain the changes in protein expression after PBNPs administration. To identify changes at the transcriptional level, we used RNA sequencing and found some related pathways, such as Ras, p53, PI3K‐Akt, and AMPK. The activation of these pathways may eventually induce mRNA transcription of antioxidant enzymes, as reported in the literature.^[^ ^30^ ^]^ However, the molecular mechanism network related to anti‐ROS is not directly related to PBNPs. Subsequently, we found that NRF2, one of the key transcriptional promoters regulating *Sod1* expression,^[^ ^22^ ^]^ did not change significantly in NPCs treated with PBNPs. Therefore, we believe that the significant increase in *Sod1* mRNA expression after PBNPs treatment was not related to NRF2, suggesting the involvement of other pathways. Therefore, this pathway was not the focus of this study.

Protein modification after transcription and translation, especially protein degradation caused by modification, is known to seriously affect protein expression. Therefore, we used CHX to inhibit protein synthesis, MG132 to inhibit the proteasome, and Chiq to inhibit lysosomes. Some results were unexpected: PBNPs could mediate the degradation of SIRT1 through the proteasome and the ubiquitination modification of SOD1 to prevent its degradation through the proteasome. In cells, we verified that PBNPs could colocalize with SOD1 and significantly improve its expression. Combined with the results of MitoTracker and TEM, we hypothesize that this process occurs outside the mitochondria. Nevertheless, it can still affect the function and structure of mitochondria, including increasing the size of mitochondria and the density of mitochondrial crista, thereby improving mitochondrial function. Overall, these results indicate that PBNPs have many effects on intracellular OS. The key regulatory mechanism is SOD1 expression. First, PBNPs promote *Sod1* mRNA expression. More importantly, PBNPs inhibit the ubiquitination modification of SOD1 to inhibit SOD1 degradation through the proteasome. The combined action of these two aspects greatly increases the expression and enzyme activity of SOD1 in cells to activate the function of mitochondria and produce NPCs in the reducing environment of the effective metabolism of OS.

Furthermore, we used the classic rat caudal puncture degeneration model, in which the initial cause of IVDD was ROS. After modeling, the IVD showed significant degeneration, accompanied by the downregulation of SOD1 expression, indicating that ROS participated in the process of NPCs degeneration, and that the downregulation of SOD1 expression may play a key role. The expression of SOD1 increased significantly after PBNPs administration, and it may be a key protein for saving and alleviating ROS in NPCs. The colocalization of the functional mitochondrial marker ATP5H^[^ ^25^ ^]^ and SOD1 also showed that the decrease in SOD1 mainly existed in or around mitochondria during the progression of degeneration. At the same time, with the addition of PBNPs, the link between SOD1 and ubiquitination decreased, the expression of SOD1 increased, and the IVD showed the rescued phenotype. This also supports the cellular experiment results: the OS of NPCs depletes mitochondria‐related SOD1 and PBNPs can improve the stability of SOD1 around mitochondria by decreasing the SOD1‐ubiquitination combination, allowing mitochondria to resist OS and delaying cell degeneration.

**Figure 8:**
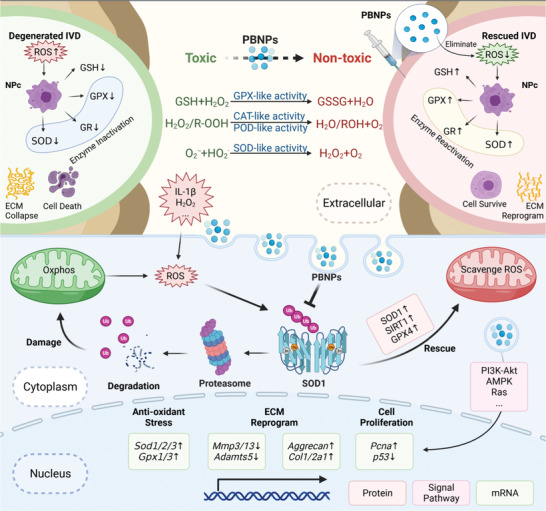
Molecular mechanism Diagram of PBNPs alleviate OS in NPCs.

Therefore, we can summarize the overall mechanism by which PBNPs affect the redox environment in NPCs as described in **Figure** **8**. The mechanism is divided into three levels. First, antioxidant enzyme‐like PBNPs can directly scavenge extracellular ROS; second, PBNPs can inhibit the ubiquitin‐proteasome degradation of SOD1 in cells, increase the number of mitochondria‐related SOD1 protein and enzyme activity, and eliminate intracellular ROS; third, PBNPs can activate Ras, p53, PI3K Akt, AMPK, and other pathways and directly affect the transcription of antioxidant enzymes (SOD, GPX, and SIRT). Under these three key mechanisms of anti‐OS, PBNPs can eventually improve the function of mitochondria in cells, improve the antioxidant capacity inside and outside NPCs, and maintain cells in a reducing environment. This further activates the viability of cells, improves the anabolic ability of cells, and reconstructs the ECM of the nucleus pulposus, which rescues ROS‐mediated IVD degeneration in animal models.

PBNPs have been widely used as cancer therapeutic contrast agents and biosensors because of their excellent magnetic capability.^[^ ^31^ ^]^ In the present study, we verified that PBNPs can display high signals on magnetic resonance T1 images, decrease T1 relaxation time and increase T1 relaxation rate, which are concentration dependent. As so, we studied and discussed the possible therapeutic effects of PBNPs on IVDD. We hope that this study will promote clinical research and the transformation of PBNPs in spinal discography. Therefore, we also improved the toxicity study of local injection of PBNPs into the caudal vertebra of rats and on key organs and obtained negative results. Nevertheless, more detailed research is needed, such as how PBNPs activate RAS, p53, PI3K Akt, AMPK, and other pathways, which key transcripts of regulated *Sod*, *Gpx*, and *Sirt* mRNA are released, whether PBNPs and SOD1 bind to each other and at which binding sites, and what is the fundamental mechanism by which PBNPs affect the ubiquitination modification of SOD1. The metabolic process of PBNPs both in cells and in vivo is crucial for MRI discography. This research group will further study these key issues and provide basic research data for clinical improvement.

## 4 Experimental Section

### Preparation of PBNPs

PBNPs were prepared according to the literature. Typically, PVP (8.0 g) (Sigma‐Aldrich, St. Louis, MO, USA) was added to an HCl solution (1 m, 50 mL) (Adamas‐beta, Shanghai, China), followed by the addition of 700 mg potassium hexacyanoferrate(III) (K_3_[Fe(CN)_6_], Adamas‐beta). The mixed solution was stirred for 1 h and then placed in an electric oven (100 °C) for 20 h. Finally, PBNPs were obtained after centrifugation and dispersed in deionized water for further use.

### Depletion of H_2_O_2_, •OH, and O_2_^•−^ by PBNPs

The H_2_O_2_ depletion capacity of PBNPs (0, 50, 100, and 500 ng mL^−1^) was explored using a colorimetric titanium sulfate assay kit (Solarbio Life Sciences, Beijing, China) according to the manufacturer's instructions. The Fe^2+^/ H_2_O_2_ system was used to generate •OH. The ability of PBNPs (0, 10, and 50 µg mL^−1^) for quenching •OH was investigated by EPR using DMPO (Adamas‐beta) as the spin probe. The xanthine/XO system was used to generate O_2_^•−^. The ability of PBNPs (0, 10, and 50 µg mL^–1^) for quenching O_2_^•−^ (SOD‐like activity) was investigated by EPR using DMPO as the O_2_^•−^ trapping agent.

### CAT, POD, and GPX‐Like Activity of PBNPs

A disodium terephthalate (TPA‐Na)/2‐hydroxyterephthalic acid (TPA‐OH) system (Adamas‐beta) was used. The CAT‐like activity of PBNPs was evaluated by assessing the inhibition of fluorescent TPA‐OH generation. Typically, TPA‐Na (10 × 10^−3^ m) was added to H_2_O_2_ (5 × 10^−3^ m) in the presence or absence of PBNPs. The mixture was shaken at 45 °C for 10 min in the dark. The sample was then withdrawn and measured using a fluorescence spectrometer (425 nm).

The POD‐like activity of the prepared PBNPs was investigated using *o*‐phenylenediamine (OPD, Adamas‐beta) as the substrate in the presence of H_2_O_2_. In brief, OPD (500 × 10^−6^ m), H_2_O_2_ (1 × 10^−3^ m), and PBNPs (50 µg mL^−1^) were mixed at room temperature. The color change of the reaction mixture was recorded at a certain reaction time. Additionally, the absorption at 442 nm over time was monitored using UV–Visible spectroscopy.

Total and selenium‐core GPX activities were assayed using the total GPX assay kit with NADPH (Beyotime, Nantong, China) and cellular GPX assay kit with NADPH (Beyotime) according to the manufacturer's instructions.

### Culture of NPCs and 293T Cell Lines

The rat NPCs used here were immortalized cell lines kindly gifted by Dr. Chen Di at the Department of Orthopedic Surgery, Rush University Medical Center (Chicago, IL, USA).^[^ ^32^ ^]^ 293T cell lines were obtained from the National Collection of Authenticated Cell Cultures. Cells were maintained in Dulbecco's modified Eagle's medium (DMEM) supplemented with 10% fetal bovine serum (FBS) and 1% penicillin–streptomycin (Gibco, Thermo Fisher Scientific, Waltham, MA, USA).

### Cell Viability Analysis

Cell viability following PBNPs treatment was evaluated using the CCK8 assay (Dojindo Laboratories Co., Ltd., Kumamoto, Japan). Cells were seeded onto 96‐well plates at a density of 3 × 10^3^ cells well^−1^ and treated with increasing concentrations of PBNPs (0.39, 0.78, 1.56, 3.12, 6.25, 12.5, 25, 50, and 100 µg mL^−1^, dissolved in DMEM with 10% FBS and 1% penicillin/streptomycin) for 24 h. After this period, the cells were incubated with fresh complete medium containing 10 µL of CCK8 reagent for 2 h at 37 °C. Complete medium containing CCK8 reagent but no cells or untreated cells was used as blank or the control group, respectively. The absorbance was measured as optical density (OD) at 450 nm using an Infinite M200 Pro multimode microplate reader (Tecan Life Sciences, Männedorf, Switzerland). The ODs of all groups were normalized to the corresponding blank ODs to account for background interference.

### High Density Culture

Approximately 120 000 NPCs were resuspended in 15 µL of MEM/F12 medium (HyClone Laboratories Inc., Logan, UT, USA) and seeded as micromasses at the bottom of a 12‐well plate to assess the ECM secretion ability of NPCs. The NPCs were attached to the bottom for 1 h at 37 °C. MEM/F12 medium (1 mL) containing 10 ng mL^−1^ insulin transferrin selenium (ITS) and 2% FBS was added. The medium was refreshed every other day. After 5 days, the micromasses were stained with Alcian blue.

### Assessment of ROS by DCFH‐DA

Assessment of ROS was performed using the Reactive Oxygen Species Assay Kit (Beyotime, Nantong, China) according to the manufacturer's protocol. Green fluorescence indicated that cells stained with DCF could be photographed by fluorescence microscopy and counted by IOD/area.

The effects of PBNPs were evaluated by flow cytometry following staining with DCFH‐DA for ROS assessment. Cell suspensions were subjected to flow cytometry on a FACSCalibur flow cytometer (BD Biosciences, Franklin Lakes, NJ, USA), counting at least 10 000 events. The DCF intensity was quantified based on the intensity of the FITC channel (488 nm excitation wavelength and 525 nm emission wavelength) on the right (Q2; positive staining for DCF) of the flow cytometric scatterplot.

### Activity of Intracellular SOD(Cu/Zn, Mn), GPX, and GR and Content of Intracellular GSH and NADP+/NADPH

Before the assay, one million NPCs were mixed with 200 µL lysis buffer and protein content was detected using the BCA Protein Assay Kit (Beyotime). The activity of SOD(Mn) was assayed using the Cu/Zn‐SOD and Mn‐SOD Assay Kit with WST‐8 (Beyotime). Intracellular GPX (total and selenium‐core) activities were assayed using the total GPX assay kit with NADPH (Beyotime) and cellular GPX assay kit with NADPH (Beyotime). GR was assayed using the GR assay kit with DTNB (Beyotime).

GSH and GSSG contents were assayed using a GSH and GSSG assay kit (Beyotime) and the protocol from the manufacturer. The GSH (µg mg^−1^) of cell weight (mg) = 2 × (total GSSG‐GSSG) (µg) / cell weight (mg) was statistically analyzed.

NADP^+^/NADPH was assayed by the NADP^+^/NADPH assay kit with WST‐8 (Beyotime).

### Western Blot

The NPCs were washed thrice with PBS. The RIPA lysis buffer with phosphatase and protease inhibitors (Thermo Fisher Scientific) was used to lyse cells for preparing protein homogenates. Equal quantities of extracted proteins (20–30 µg) were resolved on 2–20% SDS‐PAGE gels, and separated proteins were electroblotted onto 0.22‐µm PVDF membranes (Merck‐Millipore, Burlington, MA, USA). The membranes were blocked with 5% BSA‐PBS at 20–25 ℃ for 1 h and then incubated with primary antibodies (diluted 1:1000 in 5% BSA‐PBS) overnight (at least for 16 h) at 4 °C. The primary antibodies used were rabbit anti‐SIRT1 (Cell Signaling Technology, CST, Danvers, MA, USA), anti‐SIRT3 (CST), anti‐SOD1 (CST), anti‐SOD2 (CST), anti‐GPX1 (Absin, Shanghai, China), anti‐GPX3 (NBP1, Colorado, USA), anti‐GPX4 (CST), mouse monoclonal anti‐B‐actin (Affinity, Jiangsu, China), and mouse monoclonal anti‐phospho‐Flag (Sigma‐Aldrich). Tris with Tween‐20 buffer salt solution (TBST) was used to wash the membranes thrice, and the membranes were incubated with anti‐rabbit or anti‐mouse immunoglobulin (Ig)G conjugated with IRDye 800CW (CST) for 1 h at 20–25 ℃. After washing the membranes thrice, immunoreactive bands were detected using the Odyssey infrared imaging system (LI‐COR, Lincoln, NE, USA). Positive immunoreactive bands were quantified using Image‐Pro Plus 6.0 (IPP6.0, Media Cybernetics Inc., Rockville, MD, USA) and normalized to *β*‐actin.

### Quantitative PCR and RNA Sequencing

A total RNA extraction kit (Axygen, Sigma‐Aldrich) was used to extract total RNA from the NPCs. The first strand complementary DNA (cDNA) synthesis kit (TAKARA, Beijing, China) was used to reverse transcribe the total RNA to first strand cDNA. Quantitative PCR was performed using the TB‐Green premix Ex Taq kit (TAKARA) system and a QuantStudio 6 Flex real‐time system (Thermo Fisher Scientific). The 2^^(−ΔΔCt)^ and 2^^(−ΔCt)^ methods were used to calculate relative gene expression. *β*‐actin and GAPDH were used as internal references. Primer sequences were designed using BLAST (NCBI, Bethesda, USA).

Extracted RNA was assayed via RNA (transcriptome) sequencing by Wuhan Huada Gene Technology Co., Ltd. (China) and analyzed for the Kyoto Encyclopedia of Genes and Genomes (KEGG) pathways, Gene Ontology (GO) cellular components, and mRNA relative expression using fragments per kilobase million (FPKM) on the Mybgi platform (Wuhan Huada Gene Technology, https://mybgi.bgi.com/tech/login).

### Co‐Immunoprecipitation

Plv‐Control (plv‐c), Flag‐SOD1, and Myc‐UBB overexpressing 293T cells cultured on a 10 cm dish were washed thrice with PBS and dissolved in 1 mL NETT buffer (0.15 m NaCl + 0.1 × 10^−3^ m EDTA + 50 × 10^−3^ m Tris‐HCl + 1% Triton X‐100) with 1 × 10^−3^ m PMSF (Beyotime) and phosphatase and protease inhibitors (Thermo Fisher Scientific) to prepare protein homogenates. To each 800 µL sample taken, 30 µL Flag magnetic beads were added and the mixture was incubated at 4 °C in a rocking bed overnight. After this period, 200 µL samples were taken and 50 µL loading buffer was added to each sample. After boiling at 95 °C for 10 min, the supernatant was removed and adsorbed onto the magnetic beads. TBST was used thrice to clean the magnetic beads. RIPA lysis buffer containing phosphatase and protease inhibitors was used to elute the protein from the beads at 95 °C for 10 min. Supernatant proteins were separated by 2–20% SDS‐PAGE.

### Histology and Immunofluorescence Staining

Intervertebral discs were cut into 5 µm sections and attached to the thickness of a glass sheet using neutral resin to seal them. For histological assessment, the paraffin tissue sections were processed for hematoxylin–eosin (HE) and Safranin O‐Fast Green staining in accordance with standard laboratory protocols. For immunofluorescence assessment, NPCs were cultured on a slide with a confluence of 10% and fixed with 4% paraformaldehyde (PFA). These cell or tissue slides were deparaffinized in graded xylene, rehydrated in graded alcohol solutions, and then incubated in antigen retrieval buffer (Roche, Basel, Switzerland) at 37 °C for 30 min. After cooling to room temperature, the slides were immersed in PBS (pH 7.4) and washed thrice for 5 min each. An auto‐fluorescence quencher was added to the sections for 5 min and blocked with blocking buffer for 30 min at room temperature. The sections were subsequently incubated with primary antibodies in a wet box at 4 °C overnight.

MitoTracker was used according to the protocol from Beyotime (MitoTracker Red CMXRos, Beyotime). Primary antibodies were used at 1:100 dilution and included rabbit anti‐COL2A1 (Affinity), anti‐Aggrecan (Affinity), anti‐SOD1 (CST), anti‐SIRT1 (CST), anti‐GPX1 (Absin), anti‐Rab5 (Abcam, Cambridge, UK), anti‐ATP5H (Abcam), anti‐Ubiquitin (CST). The next day, the sections were washed with PBS and then incubated with Alexa Fluor 594 and 647 conjugated secondary antibody (anti‐rabbit, 1:500; CST) for 50 min at room temperature in the dark. The sections were washed with PBS and incubated with DAPI solution (Sigma‐Aldrich) for 10 min in the dark to stain the cell nuclei. The sections were subjected to final PBS washes, air‐dried, and then sealed with anti‐fluorescence‐quenching tablets. Digital fluorescence images were captured using a confocal microscope (Leica Microsystems, Wetzlar, Germany), and IOD measurements were performed using IPP6.0.

### Animals and Surgical Procedures

All animal experiments were approved by the Institutional Animal Care and Ethics Committee of the Ninth People's Hospital, Shanghai Jiaotong University School of Medicine (Shanghai, China; Ethics Number: SH9H‐2021‐TK326‐1) and performed in accordance with the principles and procedures of the National Institutes of Health (NIH) Guide for the Care and Use of Laboratory Animals and the Guidelines for Animal Treatment of Shanghai Jiaotong University. Eight‐week‐old male Sprague–Dawley rats (Shanghai Lab, Animal Research Center Co. Ltd., Shanghai, China) were housed under pathogen‐free conditions at 26–28 °C and 50–65% humidity with a 12‐h day/night cycle. Before surgical procedures, rats were anesthetized by intraperitoneal injection of pentobarbital sodium (5 mg/100 g body weight). The tails were sterilized with iodinated polyvinylpyrrolidone, and a ventral longitudinal skin incision was made over the tail to reveal the IVD at the coccyx vertebrae (Co) 3–7. The IVDs at Co6/7 were used as sham controls, and those at Co3/4, Co4/5, and Co5/6 were used as the experimental groups. IVDs were punctured with a 20‐gauge sterile needle oriented perpendicular to the skin to ensure insertion at the center of the disc level through the annulus fibrosus (AF), into the nucleus pulposus (NP), and rotating the needle once. The incision was then sutured, and the rats were allowed to recover for 2 weeks. Surgical exposure of the IVDs at Co3/4, Co4/5, Co5/6, and Co6/7 was repeated, and 5 µL of PBNPs (2 and 0.2 µg) were injected into each IVD. The incision was sutured, and the rats were allowed to recover for 4 weeks. At the end of the experimental period, all remaining rats were sacrificed, and the tails were extracted, cleaned of soft tissues, and the vertebral column was fixed in 4% PFA.

### Radiographic and MRI Analysis

Digital X‐ray imaging of the punctured IVDs was conducted in the anteroposterior axis with a 21 lp mm^–1^ detector that provided up to ×5 geometric magnification (Faxitron VersaVision; Faxitron Bioptics LLC, Tucson, AZ, USA). MRI imaging of the same punctured IVDs was carried out on a Siemens Magnetom Prisma 3.0T (Siemens Healthineers, Erlangen, Germany) with the following parameters: TR 2400 ms, TE 3.4 ms, 0.7 × 10^−3^ m thickness, FOV 226 × 226 mm.

The parameters of T1 mapping were changed to TR 7 ms, TE 2.49 ms, FOV 160 × 160 mm. T1 relaxation time and T1 relaxation rate of gradient concentrations of PBNPs and Gd‐DTPA were tested by T1 mapping.

### Statistical Analyses

All experiments were repeated three to six times. The specific number of repetitions is shown in the statistical chart and figure legends of the related experiments. Preprocessing of data included transformation, normalization, and evaluation of outliers, when necessary. Data from all experiments are reported as the mean ± standard deviation (SD). The significance of differences between groups was tested with Student's *t*‐test or one‐way analysis of variance (ANOVA) using SPSS 19.0 (IBM Corporation, Armonk, NY, USA). The difference was considered significant if the *p*‐value was less than 0.05 (* indicates *p* < 0.05, ** indicates *p* < 0.01, *** indicates *p* < 0.001, **** indicates *p* < 0.0001).

## Conflict of Interest

The authors declare no conflict of interest.

## Supporting information

Supporting Information

## Data Availability

The data that support the findings of this study are available in the supplementary material of this article.
