# Supplementary material for: Prussian Blue Nanoparticles Stabilize SOD1 from Ubiquitination‐Proteasome Degradation to Rescue Intervertebral Disc Degeneration
Source: Adv Sci (Weinh). 2022 Feb 6;9(10):2105466. doi: 10.1002/advs.202105466 (PMC8981911; doi:10.1002/advs.202105466)
Supplement: Supplementary file 1 — Supporting Information [file ADVS-9-2105466-s001.pdf]

ADVANCED SCIENCE
Open Access

Supporting Information

for *Adv. Sci.*, DOI 10.1002/advs.202105466

Prussian Blue Nanoparticles Stabilize SOD1 from Ubiquitination-Proteasome Degradation to Rescue Intervertebral Disc Degeneration

Tangjun Zhou, Xiao Yang, Zhiqian Chen, Yangzi Yang, Xin Wang, Xiankun Cao, Chen Chen, Chen Han, Haijun Tian, An Qin, Jingke Fu* and Jie Zhao**

ADVANCED SCIENCE
Open Access

Supporting Information

for *Adv. Sci.*, DOI: 10.1002/advs.202105466

Prussian Blue Nanoparticles Stabilize SOD1 from Ubiquitination-proteasome Degradation to Rescue Intervertebral Disc Degeneration

Tangjun Zhou, Xiao Yang, Zhiqian Chen, Yangzi Yang, Xin Wang, Xiankun Cao, Chen Chen, Chen Han, Haijun Tian, An Qin, Jingke Fu*, and Jie Zhao**

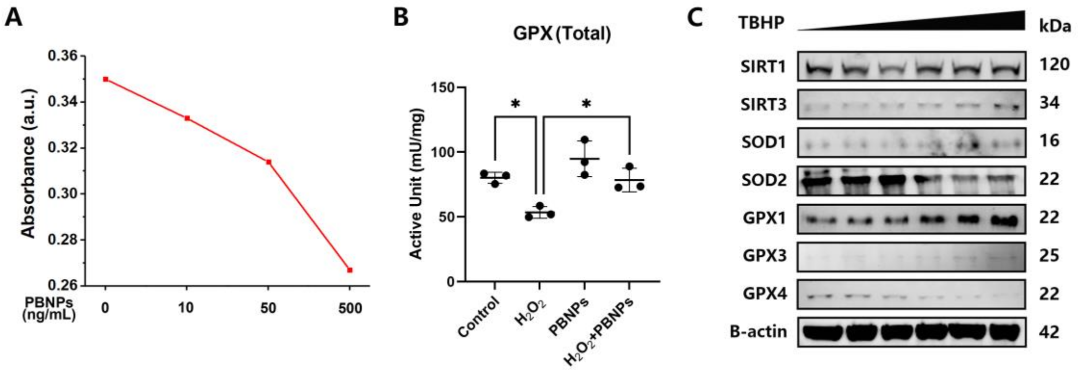

Figure S1.

(A) The absorbance change of the H_2O_2-titanium sulfate reaction system at 415 nm upon the adding of different concentrations of PBNPs, demonstrating the PBNPs concentration dependent depletion of H_2O_2.

(B) Statistical chart shows active unit (mU/mg) of GPX(Total) after H_2O_2 (0.6×10^{-3}%) stimulation and PBNPs (2μg/ml) treatment via Total Glutathione Peroxidase Assay Kit with NADPH.

(C) Expression of SIRT1, SIRT3, SOD1, SOD2, GPX1, GPX3, GPX4, B-actin protein from gradient concentrations of TBHP (0.125-2μg/ml) treated NP cells detected by WB.

The data were presented using mean ± S.D. * indicates $p < 0.05$. The experiments above were repeated three times.

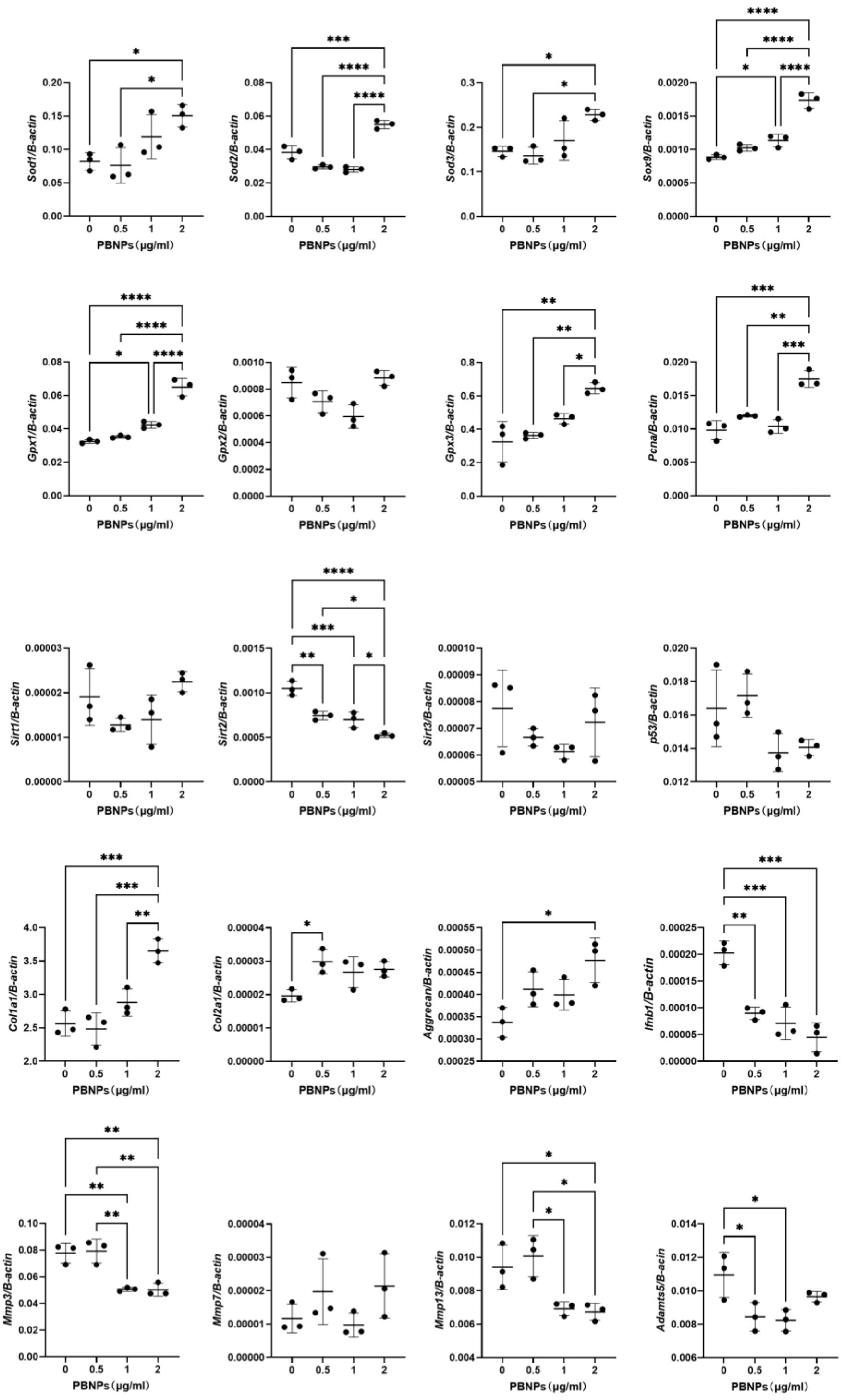

Figure S2.

Expression of *Sod1*, *Sod2*, *Sod3*, *Gpx1*, *Gpx2*, *Gpx3*, *Sox9*, *Col1a1*, *Col2a1*, *Aggrecan*, *Pcna*, *p53*, *Sirt1*, *Sirt2*, *Sirt3*, *Adamts5*, *Mmp3*, *Mmp13*, *Ifnb1* mRNA from gradient concentrations of PBNPs (0.5, 1, 2μg/ml) treated NP cells detected by PCR. Date showed as Fold Change ($2^{-\Delta CT}$).

The data were presented using mean ± S.D. * indicates $p < 0.05$, ** indicates $p < 0.01$, *** indicates $p < 0.001$, **** indicates $p < 0.0001$. The experiments above were repeated three times.

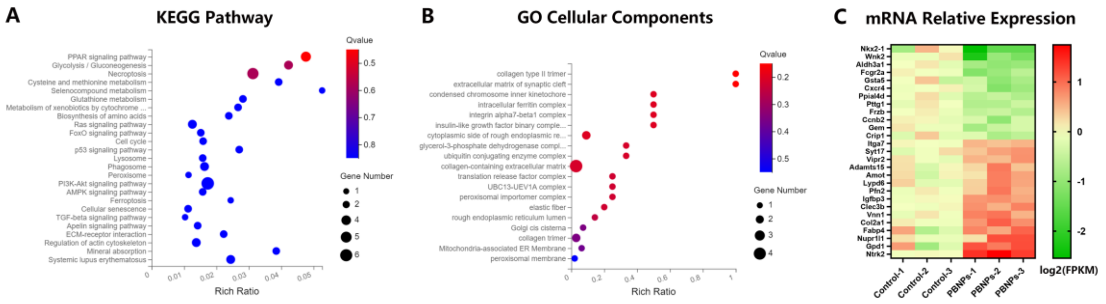

Figure S3.

(A) KEGG Pathway concluded from RNA-seq of three Control groups compared with three PBNPs (2μg/ml) groups.

(B) GO Cellular Components concluded from RNA-seq of three Control groups compared with three PBNPs (2μg/ml) groups.

(C) mRNA Relative Expression concluded from RNA-seq of three Control groups compared with three PBNPs (2μg/ml) groups.

The experiments above were repeated three times.

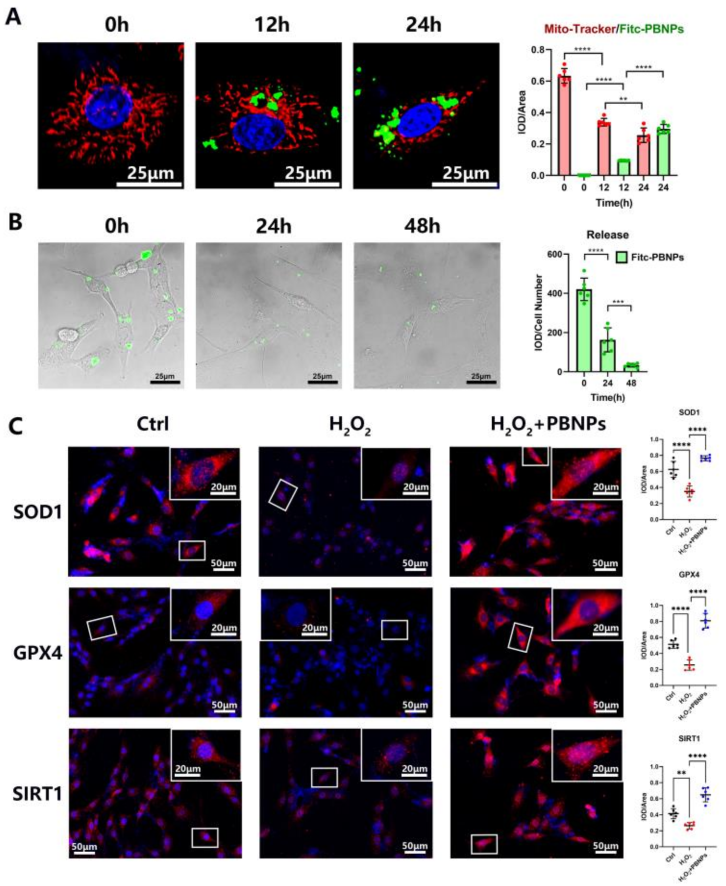

Figure S4.

(A) Fitc-PBNPs was treated to NP cells with increasing time and localization of mito-tracker with Fitc-PBNPs was detected by Confocal. Statistical chart shows IOD/Area of red and green fluorescence.

(B) Fitc-PBNPs was removed from NP cells for 24h treating. Statistical chart shows IOD/Area of

green fluorescence.

(C) Expression of SIRT1, GPX4, MMP3, SOD1 protein from H_2O_2 (0.6×10^{-3}%) and PBNPs (2μg/ml) treated NP cells detected by IHF. Statistical chart shows IOD/Area of red fluorescence.

The data were presented using mean ± S.D. ** indicates $p < 0.01$, **** indicates $p < 0.0001$. The experiments above were repeated six times.

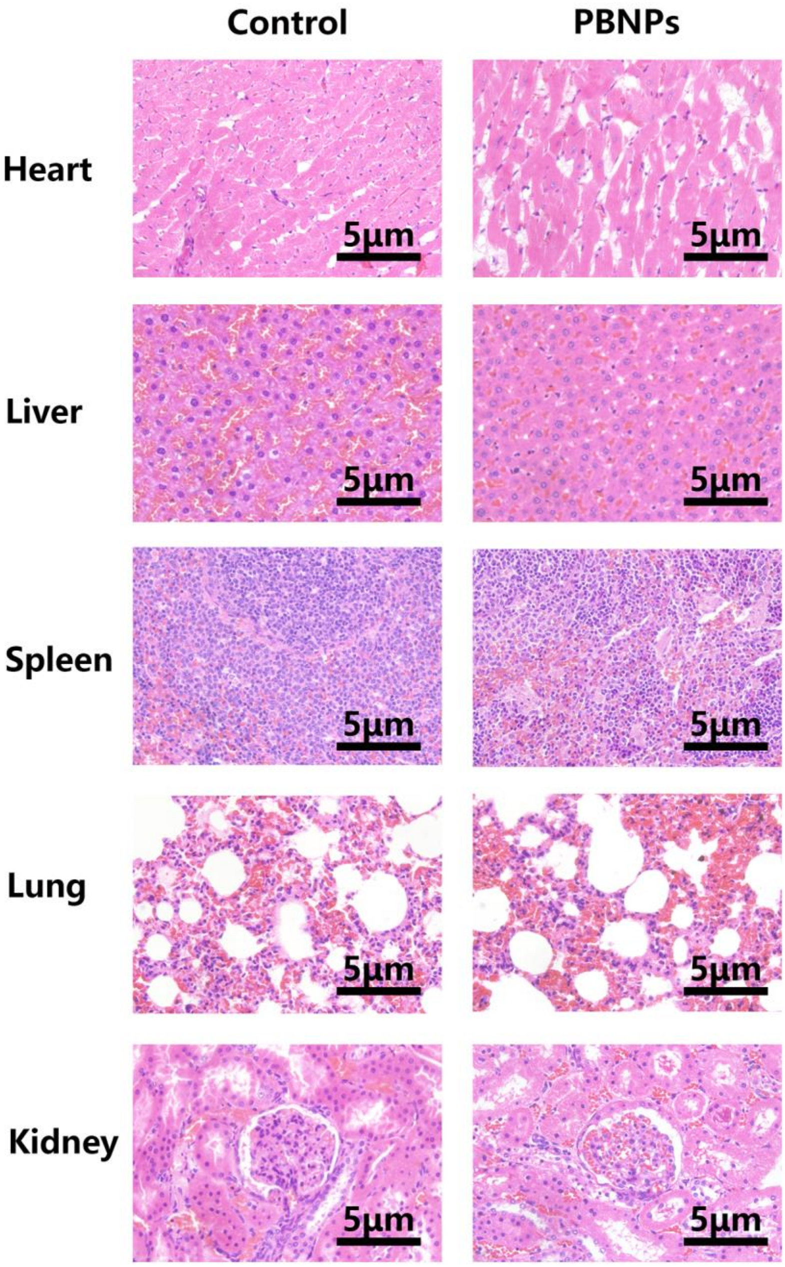

Figure S5.

HE staining of heart, liver, spleen, lung, kidney from control and PBNPs (2μg) injection group. Rats were sacrificed 4 weeks after injection. The experiments above were repeated five times.

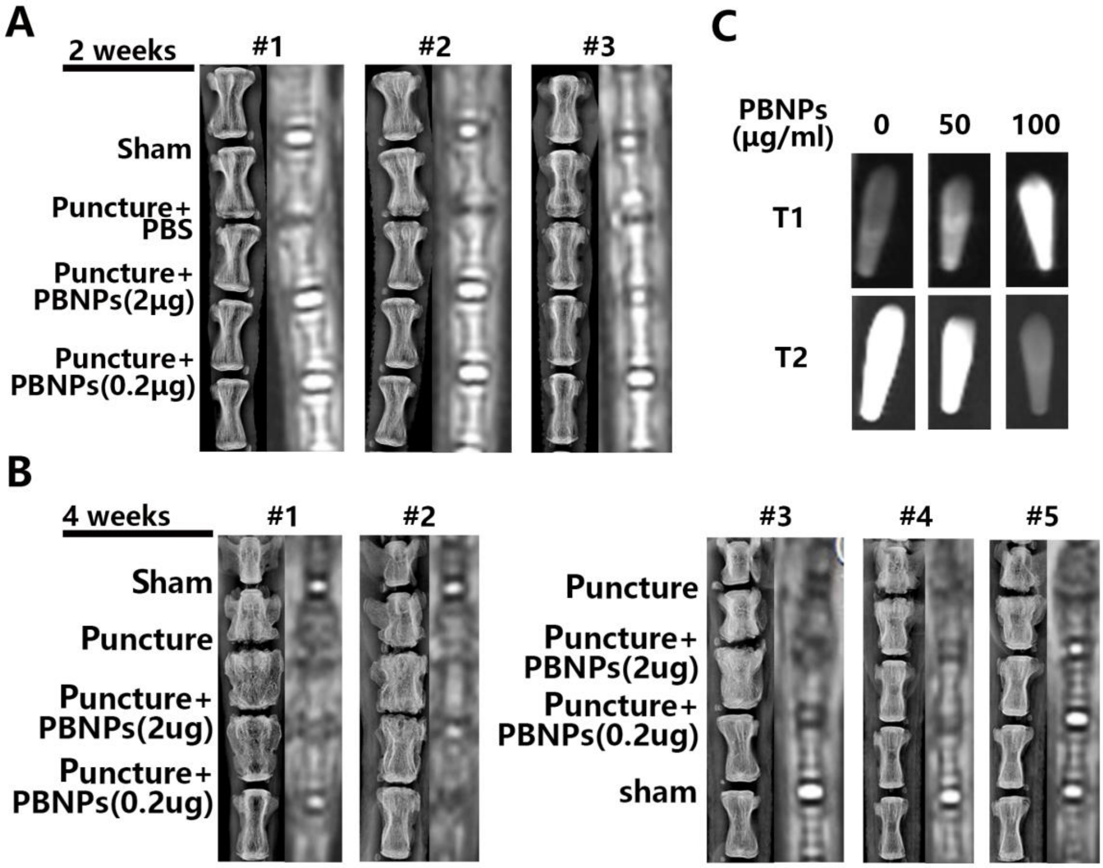

Figure S6.

(A-B) X-ray and MRI of rat tail including puncture group, puncture with PBNPs (2μg) injection group, puncture with PBNPs (0.2μg) injection group and sham group. Three rats were sacrificed 2 weeks and five rats were sacrificed 4 weeks after injection.

(C) MRI of gradient concentrations of PBNPs (0, 50, 100μg/ml) in T1 and T2 image.

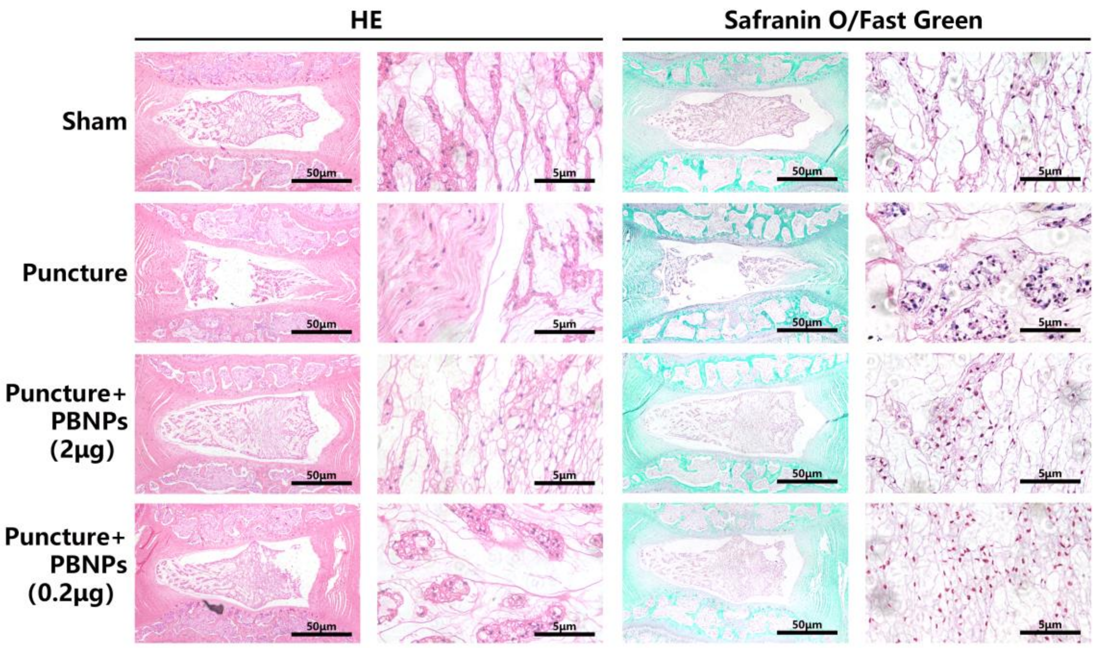

Figure S7.

HE and Safranin O-Fast Green staining of sham group, puncture group, puncture with PBNPs (2μg) injection group, puncture with PBNPs (0.2μg) injection group. Rats were sacrificed 2 weeks after injection. The experiments above were repeated three times.

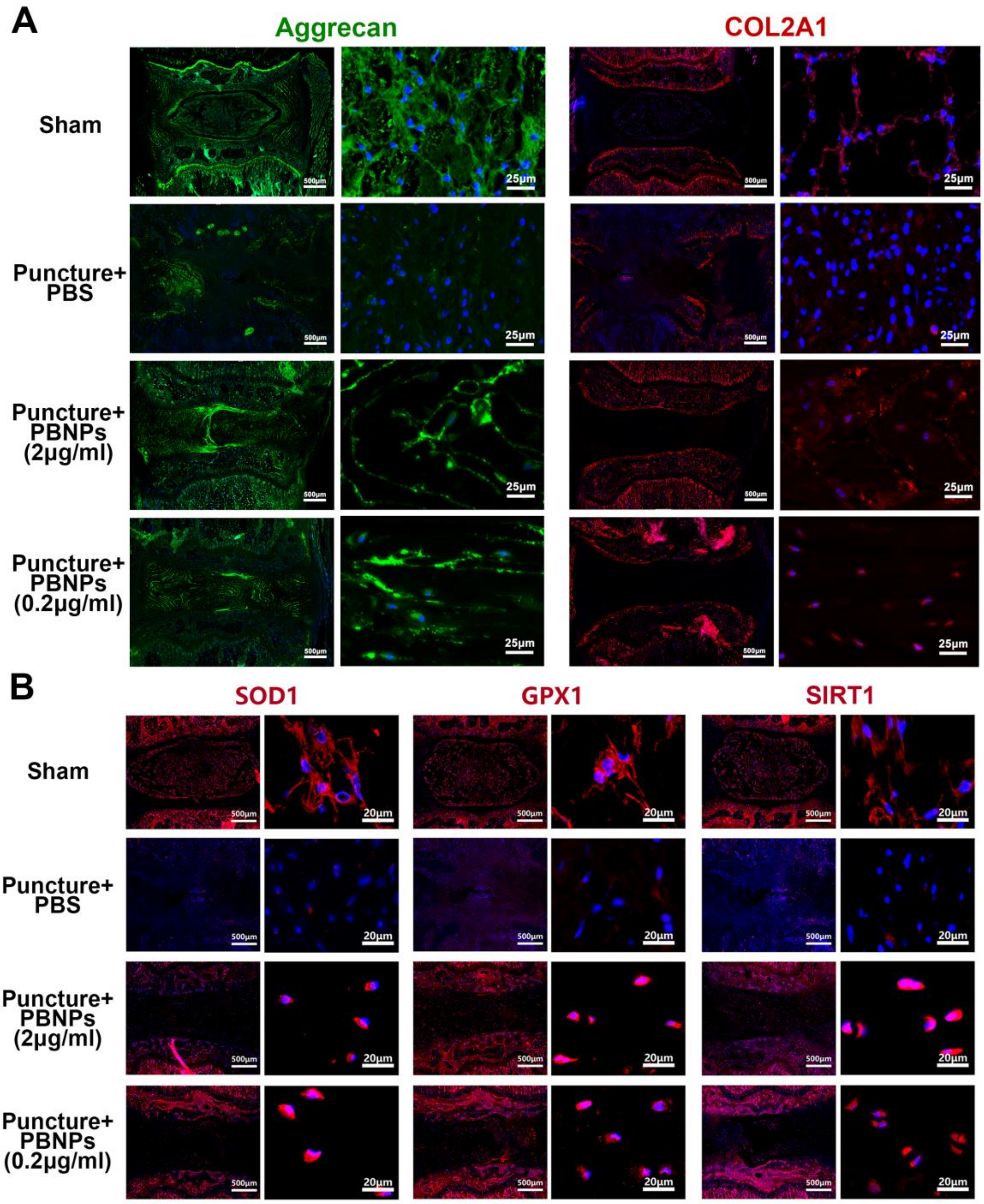

Figure S8.

(A) Aggrecan and COL2A1 in sham group, puncture group, puncture with PBNPs (2μg) injection group, puncture with PBNPs (0.2μg) injection group via IHF.

(B) SOD1, GPX1 and SIRT1 in sham group, puncture group, puncture with PBNPs (2μg) injection group, puncture with PBNPs (0.2μg) injection group via IHF.

The experiments above were repeated five times.

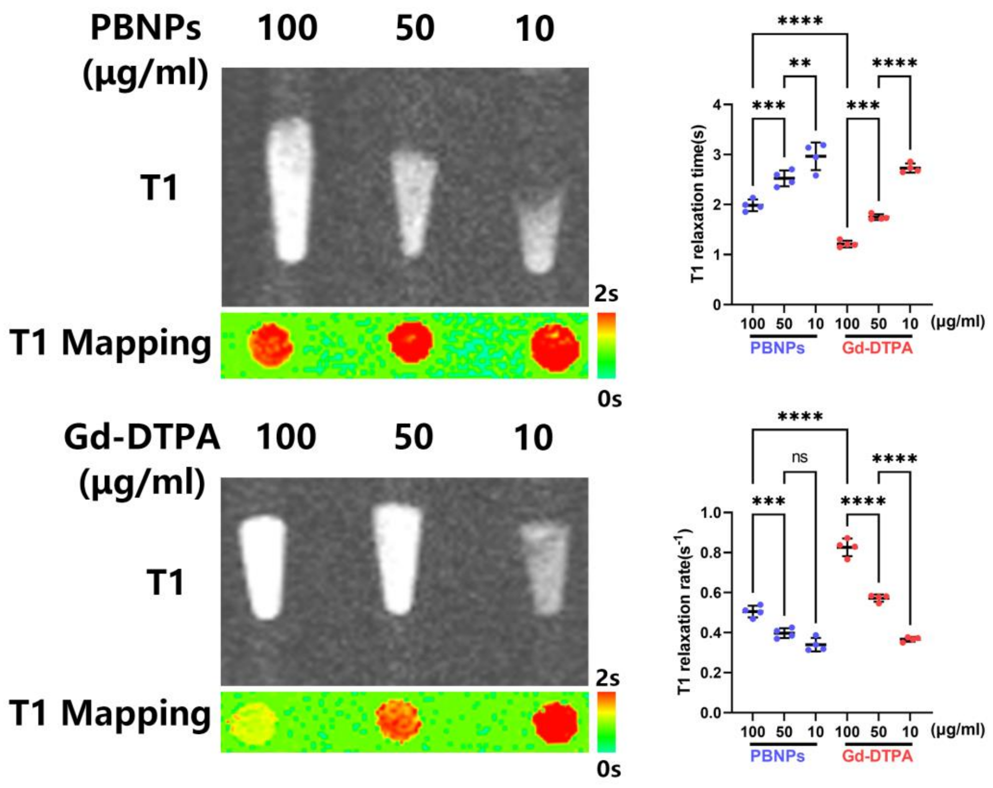

Figure S9.

MRI of gradient concentrations of PBNPs (10, 50, 100μg/ml) and Gd-DTPA (10, 50, 100μg/ml) in T1 image and T1 mapping image. The T1 relaxation time and T1 relaxation rate tested by T1 mapping image were showed in statistical chart. The data were presented using mean ± S.D. ns indicates $p > 0.05$, ** indicates $p < 0.01$, *** indicates $p < 0.001$, **** indicates $p < 0.0001$. The experiments above were repeated four times.

PCR primers information

Gene	Accession Number	Description	5'-Primer-3'
Sod1	NM_017050.1	F	TTTTGCACCTTCGTTTCCTGC
		R	ACACCACAACTGGTTCACCG
Sod2	NM_017051.2	F	AAGAAGTGAAGCCCTTCCGC
		R	AGCGTCCAAGCAATTCAAGC
Sod3	NM_012880.2	F	TCAGAGGCTCTTTCTCAGGC
		R	CGCCTCCCGTTGTTTTCCTA
Gpx1	NM_030826.4	F	AGTGCGAGGTGAATGGTGAG
		R	TCGATGTCGATGGTGCGAAA
Gpx2	NM_183403.2	F	GCATGGCTTACATCGCCAAG
		R	GGAAGCCGAGAACCACTAGG
Gpx3	NM_022525.4	F	TGTGCTCACGGTTTGGTACA
		R	GAGGCTGGTGGCATAGTGAG
Sirt1	NM_001372090.1	F	ATGGTATTTATGCTCGCCTTGC
		R	TTGCTTTCCTTCCACTGCACA
Sirt2	NM_001008368.1	F	TGAAAAACTTAGCCCTCCCTCA

		R	AGGCGACAATAGGACCCACC
Sirt3	NM_001106313.2	F	TGTGGGGTCCGGGAGTATTA
		R	TACCACTGGGTGTGCTGATG
Sox9	NM_080403.2	F	TCCCCGCAACAGATCTCCTA
		R	AGCTGTGTGTAGACGGGTTG
Col1a1	NM_053304.1	F	GGATCGACCCTAACCAAGGC
		R	GATCGGAACCTTCGCTTCCA
Col2a1	NM_053593.2	F	GTGTGACACTGGGAATGTCCT
		R	CTTGGCCCTAATTTTCCACTGGC
Aggrecan	NM_001361500.1	F	TGCAGACATTGACGAGTGCC
		R	AGAGAGTGTCCGTCAGACCA
Pcna	NM_022381.3	F	AAGGCTTCGACACATACCGC
		R	AATATGGCTAAGGTCCCGGC
p53	NM_030989.3	F	CCCCTGAAGACTGGATAACTGT
		R	CAGGAGCTGACACTTGGAGG
Adamts5	NM_011782.2	F	GAGAACCCTGCAAAACAGCC
		R	AACCATACAAGTGCCTTTTCTCT
MMP3	NM_010809.2	F	CCCTGCAACCGTGAAGAAGA
		R	GACAGCATCCACCCTTGAGT
MMP13	NM_008607.2	F	AGAAGTGTGACCCAGCCCTA
		R	GGTCACGGGATGGATGTTCA
Ifnb1	NM_019127.1	F	CAACCTCAGCTACAGGACGG
		R	TCGTGGATGTCACCCAAGTC
β-actin	NM_031144.3	F	ACCCGCGAGTACAACCTTC
		R	ATGCCGTGTTCAATGGGGTA
